# Synthesis of a High-Capacity α-Fe_2_O_3_@C Conversion Anode and a High-Voltage LiNi_0.5_Mn_1.5_O_4_ Spinel Cathode and Their Combination in a Li-Ion Battery

**DOI:** 10.1021/acsaem.1c01585

**Published:** 2021-07-26

**Authors:** Shuangying Wei, Daniele Di Lecce, Riccardo Messini D’Agostini, Jusef Hassoun

**Affiliations:** † Department of Chemical, Pharmaceutical and Agricultural Sciences, University of Ferrara, Via Fossato di Mortara, 17, 44121 Ferrara, Italy; ‡ Graphene Labs, Istituto Italiano di Tecnologia, via Morego 30, 16163 Genova, Italy; § National Interuniversity Consortium of Materials Science and Technology (INSTM), University of Ferrara Research Unit, Via Fossato di Mortara, 17, 44121 Ferrara, Italy

**Keywords:** carbon-coating, γ-Fe_2_O_3_, α-Fe_2_O_3_@C, high-voltage, LiNi_0.5_Mn_1.5_O_4_, lithium-ion battery

## Abstract

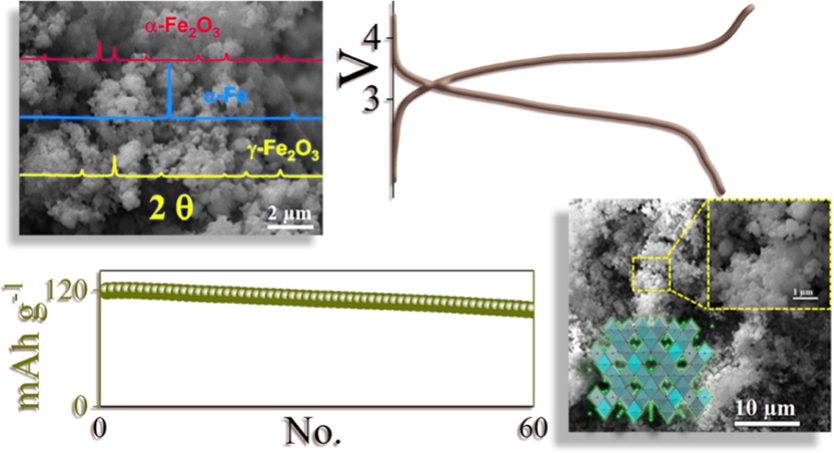

A Li-conversion α-Fe_2_O_3_@C nanocomposite anode and a high-voltage LiNi_0.5_Mn_1.5_O_4_ cathode are synthesized in parallel, characterized, and combined in a Li-ion battery. α-Fe_2_O_3_@C is prepared *via* annealing of maghemite iron oxide and sucrose under an argon atmosphere and subsequent oxidation in air. The nanocomposite exhibits a satisfactory electrochemical response in a lithium half-cell, delivering almost 900 mA h g^–1^, as well as a significantly longer cycle life and higher rate capability compared to the bare iron oxide precursor. The LiNi_0.5_Mn_1.5_O_4_ cathode, achieved using a modified co-precipitation approach, reveals a well-defined spinel structure without impurities, a sub-micrometrical morphology, and a reversible capacity of *ca.* 120 mA h g^–1^ in a lithium half-cell with an operating voltage of 4.8 V. Hence, a lithium-ion battery is assembled by coupling the α-Fe_2_O_3_@C anode with the LiNi_0.5_Mn_1.5_O_4_ cathode. This cell operates at about 3.2 V, delivering a stable capacity of 110 mA h g^–1^ (referred to the cathode mass) with a Coulombic efficiency exceeding 97%. Therefore, this cell is suggested as a promising energy storage system with expected low economic and environmental impacts.

## Introduction

Electric vehicles (EVs), hybrid-EVs (HEVs), and plug-in HEVs are predominantly powered by the most common version of the lithium-ion battery, that is, the one combining a graphite anode with a layered transition-metal-oxide cathode and employed in common portable electronics.^1−4^ This system is based on the electrochemical (de)insertion of lithium into and from the electrode materials^5^ and can typically store *ca.* 250 W h kg^–1^ for a high number of charge/discharge cycles.^4,6^ Graphite uptakes Li^+^ delivering a capacity of 372 mA h g^–1^, which is limited by the amount of alkali-metal ions stored within the carbon layers, reaching a maximum of 0.16 Li-equivalents per mole of C, that is, according to the LiC_6_ chemical formula.^7,8^ Transition-metal oxides react in the cell by an electrochemical conversion pathway mainly occurring below 1.5 V versus Li^+^/Li and involving a multiple exchange of electrons, which ensures a higher capacity than that of graphite.^9−14^ However, this intriguing class of materials intrinsically suffers from poor electrical conductivity and a large volume change throughout the electrochemical process, which causes the voltage hysteresis and rapid cell decay upon cycling.^15^ A suitable strategy to mitigate the various issues hindering the efficient use of these alternative anodes is represented by engineering nanostructured oxides with an increased active surface.^16^ Indeed, Li-conversion materials, such as CuO, NiO, and MnO, can react with lithium to form the corresponding metal (Cu, Ni, and Mn) and lithium oxide (Li_2_O), with remarkably high specific capacities of 650,^17^ 883,^18^ and 440 mA h g^–1^,^19^ respectively. Higher capacity values, that is, 910 mA h g^–1^, may be achieved using α-Fe_2_O_3_, which reacts *via* the conversion pathway leading to Fe and Li_2_O.^20,21^ For instance, nanomembranes,^22^ nanofibers,^23^ nanowires,^24^ and nanobelts^25^ of α-Fe_2_O_3_ have been synthesized to increase the cycle life and the rate capability of the cell. However, this approach was limited by the possible undesired reactions between the electrolyte and the oxide nanoparticles, which lead to capacity decay upon cycling or even to safety concerns,^26^ as well as by the relevant costs due to complex synthesis techniques.^27,28^ Highly conductive carbon matrixes incorporating α-Fe_2_O_3_ have been proposed to decrease the hysteresis of the conversion reaction^29^ and achieve *ad hoc*-designed nanostructures.^30^ Indeed, γ-Fe_2_O_3_@CNTs,^31^ Fe_2_O_3_@C flakes,^20^ and Fe_2_O_3_@C nanocomposites^32^ have shown an improved electrochemical process and excellent cycling stability. In our previous work, we have shown the cell performances of a Fe_2_O_3_–MCMB composite obtained by high-energy ball-milling, which revealed satisfactory characteristics for application in a lithium battery.^33^ Besides, a fine material tuning^31^ combined with simple synthetic approaches of iron oxide nanocomposites might facilitate practical applications and scaling up.^28,34^ We have lately adopted a facile two-step synthesis to prepare a C-coated nanostructured NiO anode successfully used in a novel lithium-ion battery using the LiNi_1/3_Co_1/3_Mn_1/3_O_2_ layered cathode.^35^ However, the voltage hysteresis and a working voltage higher than that of conventional graphite may actually jeopardize the potential use of conversion electrodes in a practical full Li-ion cell.^35^ On the other hand, high-voltage cathodes exploiting the spinel-type structure and a Li_*x*_M_*y*_N_(2–*y*)_O_4_ chemical formula (where M and N are transition metals, e.g., Ni, Mn, or Fe) appeared as the ideal candidates for enabling the use of the Li-conversion anodes.^36−39^ Among them, LiNi_0.5_Mn_1.5_O_4_ revealed the most suitable performance in the lithium cell, namely, a working voltage of 4.8 V, a theoretical capacity of 147 mA h g^–1^, and high rate capability.^40−42^ Herein, we extended the approach previously adopted for the synthesis of NiO@C^35^ to prepare a C-coated α-Fe_2_O_3_ nanocomposite anode and concomitantly prepared by the *ad hoc* developed method, a LiNi_0.5_Mn_1.5_O_4_ spinel cathode. α-Fe_2_O_3_@C has been obtained from maghemite iron oxide (γ-Fe_2_O_3_) and sucrose, by annealing under reducing conditions with subsequent oxidation at mild temperatures in air. The LiNi_0.5_Mn_1.5_O_4_ spinel cathode has been achieved using a modified co-precipitation route employing acetate and oxalic acid, leading to sub-micrometric active particles. The structure, morphology, composition, and electrochemical behavior in lithium half-cells of these two electrodes have been investigated. Subsequently, the α-Fe_2_O_3_@C anode and the LiNi_0.5_Mn_1.5_O_4_ cathode have been coupled in a new lithium-ion full-cell efficiently operating at 3.2 V.

## Experimental Section

### Synthesis of α-Fe_2_O_3_@C

3.0 g of γ-Fe_2_O_3_ (Sigma-Aldrich, 50 nm) and 3.0 g of sucrose (Sigma-Aldrich) were dispersed in a solution of water and ethanol in a 1:1 v/v ratio (50 mL) and stirred at 70 °C until solvent evaporation (*ca.* 6 h). The mixture was treated for 10 h at 120 °C in an argon atmosphere and then annealed for 3 h at 700 °C (heating rate, 5 °C min^–1^) to obtain a reduced sample (indicated as α-Fe@C), which was ground and subsequently heated in air at 380 °C for 24 h (heating rate, 5 °C min^–1^) to prepare the final composite (indicated as α-Fe_2_O_3_@C).

### Synthesis of LiNi_0.5_Mn_1.5_O_4_

LiNi_0.5_Mn_1.5_O_4_ was prepared using a modified co-precipitation pathway.^43,44^ LiCH_3_COO·2H_2_O (99%, Sigma-Aldrich), Ni(CH_3_COO)_2_·4H_2_O (99%, Sigma-Aldrich), and Mn(CH_3_COO)_2_·4H_2_O (99%, Sigma-Aldrich) were dissolved with a Li/Ni/Mn molar ratio of 1.1:0.5:1.5 in a water/ethanol mixture to get solution A (water/ethanol 1:5 v/v). Furthermore, H_2_C_2_O_4_·2H_2_O (99%, Aldrich) was dissolved in an identical hydro-alcoholic solution (B). This latter solution (B) was dropwise added to solution A with stirring of the mixture and then kept at ambient temperature for 12 h to precipitate the metal oxalates. Afterward, the precipitate was treated for 12 h at 80 °C under constant stirring to evaporate water and ethanol. The precipitate was annealed for 6 h at 500 °C in a dry air flow to obtain an oxide powder (heating ramp, 5 °C min^–1^). This powder was ground in a mortar, pressed into pellets, and calcined for 12 h at 800 °C in a dry air flow to obtain LiNi_0.5_Mn_1.5_O_4_ (heating ramp, 5 °C min^–1^).

### Characterization

X-ray diffraction (XRD) patterns of the materials were collected using a Bruker D8-Advance equipped with a Cu Kα source by changing the 2θ angle with steps of 0.02° every 10 s. Scanning electron microscopy (SEM, Zeiss EVO 40) and transmission electron microscopy (TEM, Zeiss EM 910) analyses were performed to detect the morphology and the microstructure of the powders. The sample composition was analyzed by energy-dispersive X-ray spectroscopy (EDS), employing the X-ACT Cambridge Instruments analyzer of the above-mentioned scanning electron microscope.

Electrode slurries were prepared by dispersing the active material, poly(vinylidene fluoride) (Solef 6020 PVDF) and conductive Super P carbon black (Timcal) in *N*-methyl pyrrolidone (NMP, Sigma-Aldrich), with a weight ratio between the solid components of 8:1:1. This slurry was cast on copper (for α-Fe_2_O_3_@C, α-Fe@C, and γ-Fe_2_O_3_) or aluminum (for LiNi_0.5_Mn_1.5_O_4_) foils by using a doctor blade (MTI Corporation). After NMP evaporation at *ca.* 70 °C, disks with diameters of 10 and 14 mm were cut out from the electrode foils and kept overnight under vacuum at 110 °C. The mass loadings of α-Fe_2_O_3_@C and LiNi_0.5_Mn_1.5_O_4_ over the electrodes were about 2.0 and 6.1 mg cm^–2^, respectively.

The electrochemical performance was determined in half cells that used a lithium metal disk as the counter/reference electrode and a glass fiber separator (Whatman GF/A) soaked in a 1 M electrolyte solution of LiPF_6_ in ethylene carbonate/dimethyl carbonate (EC/DMC), 1:1 (v/v). Coin-type cells (CR2032, MTI Corporation) and T-type cells were made in a glovebox (Ar atmosphere, MBraun), where H_2_O and O_2_ contents were kept below 1 ppm. The behavior of the α-Fe_2_O_3_@C electrode in the lithium cell (as well as those of α-Fe@C and γ-Fe_2_O_3_ for comparison) was investigated by cyclic voltammetry (CV) and electrochemical impedance spectroscopy (EIS, T-type cell configuration) employing a VersaSTAT MC Princeton Applied Research (PAR) multichannel potentiostat. CV data were collected within the voltage range from 0.01 to 2.8 V versus Li^+^/Li using a scan rate of 0.1 mV s^–1^. Impedance spectra were taken before cycling the cell [i.e., open circuit voltage (OCV)] as well as after the first, second, and third cycles, using an alternate voltage with an amplitude of 10 mV from a frequency of 500 kHz to a frequency of 100 mHz. Galvanostatic cycling tests were carried out to study the electrodes in coin-type, lithium half-cells, which were charged and discharged between 0.01 and 2.8 V at C/5 (for α-Fe_2_O_3_@C, α-Fe@C, and γ-Fe_2_O_3_) and between 3 and 5 V at C/2 (for LiNi_0.5_Mn_1.5_O_4_). 1 C was 1007 mA g^–1^ for α-Fe_2_O_3_@C, α-Fe@C, and γ-Fe_2_O_3_ and 147 mA g^–1^ for LiNi_0.5_Mn_1.5_O_4_. A rate capability test of α-Fe_2_O_3_@C was carried out by changing stepwise the current from C/10 to 2 C in the 0.01–2.8 V range.

A Li-ion cell was assembled coupling the α-Fe_2_O_3_@C anode with the LiNi_0.5_Mn_1.5_O_4_ cathode. Before use in the full cell, the α-Fe_2_O_3_@C electrode was electrochemically treated in a half-cell by performing three galvanostatic cycles at C/5 between 0.01 and 2.8 V, with the last cycle ending at 2.1 V, in order to ensure a steady working condition of the anode in the lithium-ion cell. The α-Fe_2_O_3_@C/LiNi_0.5_Mn_1.5_O_4_ battery was made using a ratio of about 2.2 between negative and positive electrodes (i.e., N/P ratio), as determined by taking into account the theoretical capacities of α-Fe_2_O_3_@C (1007 mA h g^–1^) and LiNi_0.5_Mn_1.5_O_4_ (147 mA h g^–1^) along with their mass loadings (2.0 and 6.1 mg cm^–2^, respectively). This full cell was charged and discharged at a C/2 rate in the 1.5–4.5 V voltage range (where 1 C is 147 mA g^–1^, as referred to the cathode mass). All galvanostatic measurements were conducted at room temperature (25 °C) with a Maccor Series 4000 battery tester.

## Results and Discussion

**Figure 1:**
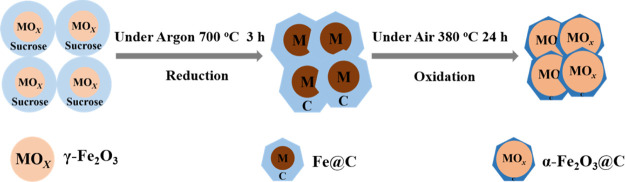
Illustration of the synthetic steps of α-Fe_2_O_3_@C (see details in the Experimental Section).

Figure 1 reports in a diagram the synthetic steps employed to prepare the α-Fe_2_O_3_@C nanocomposite (see details in the Experimental Section). The synthesis involves the reduction of pristine γ-Fe_2_O_3_ with sucrose at 700 °C under argon to form an α-Fe core and a carbon shell, and subsequent oxidation to α-Fe_2_O_3_@C at 380 °C under air. This pathway, previously adopted for achieving a NiO@C electrode, leads to carbon-coated metal oxide particles suitable for battery application and includes simple experimental steps.^35^ In addition, pristine γ-Fe_2_O_3_ and sucrose are cheap and widely available precursors, thus possibly providing a scalable two-step method with a moderate economic impact to prepare an efficient and alternative iron oxide anode for use in Li-ion batteries.^28^

**Figure 2:**
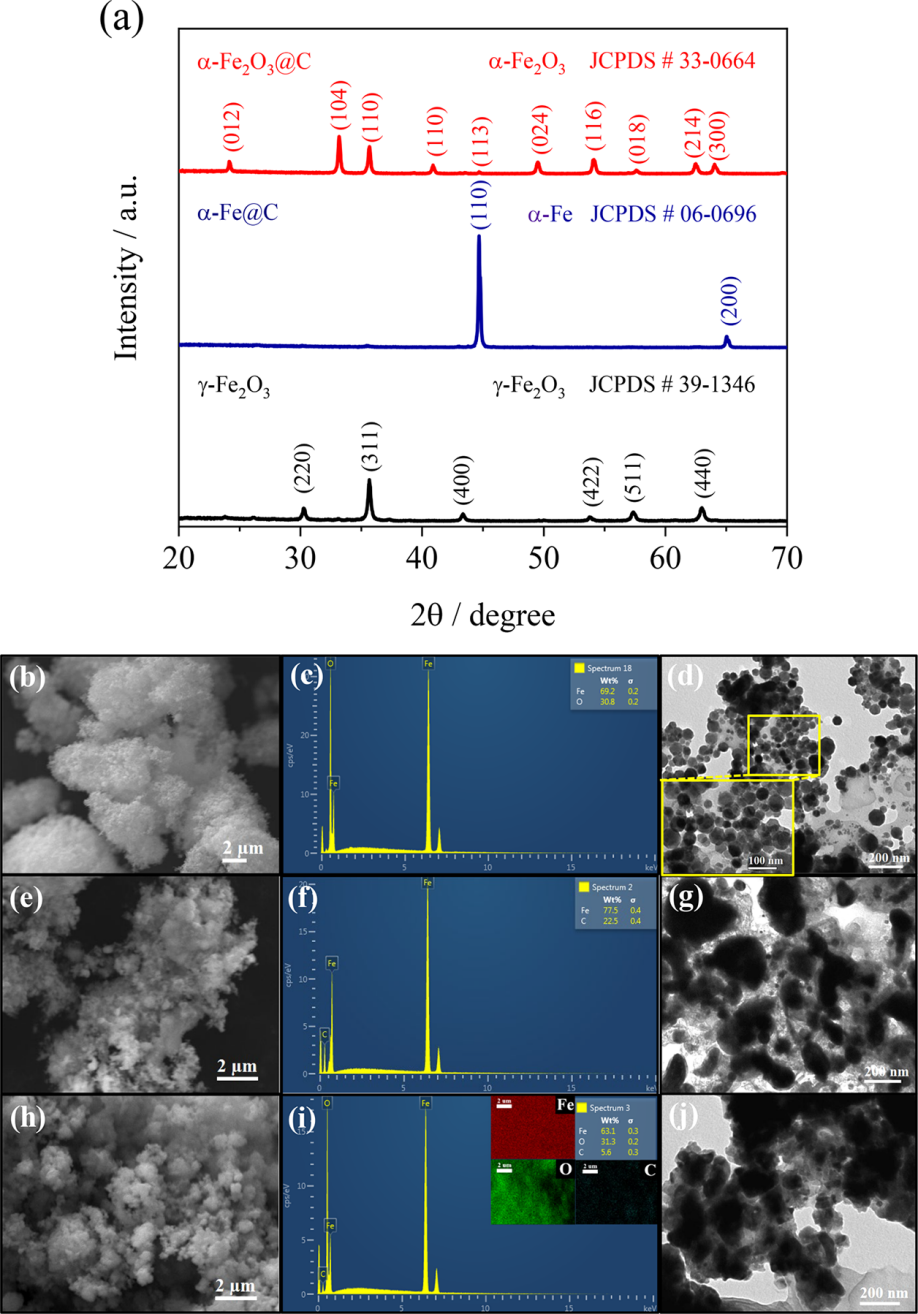
(a) XRD patterns of γ-Fe_2_O_3_, α-Fe@C, and α-Fe_2_O_3_@C. (b,e,h) SEM images, (c,f,i) EDS spectra, and (d,g,j) TEM images of γ-Fe_2_O_3_ (b–d), α-Fe@C (e–g), and α-Fe_2_O_3_@C (h–j); inset in (i) represents the elemental mapping images of Fe, O, and C of α-Fe_2_O_3_@C.

The structure, morphology, and elemental composition of the α-Fe_2_O_3_@C material are provided in Figure 2. The XRD patterns of Figure 2a indicate a cubic structure both for the precursor (γ-Fe_2_O_3_, JCPDS # 39-1346) and for the synthesis intermediate (α-Fe, JCPDS # 06-0696), while the final material shows a hexagonal arrangement (α-Fe_2_O_3_, JCPDS # 33-0664). The above reflections reveal a substantial change of phase and crystal structure during the synthesis steps; indeed, the data suggest an almost complete reduction of the pristine maghemite (γ-Fe_2_O_3_) to metallic iron (α-Fe) by annealing under argon in the presence of sucrose, and a subsequent oxidation to hematite (α-Fe_2_O_3_) upon mild treatment under air. Therefore, the pure cubic phase of γ-Fe_2_O_3_ converts into α-Fe due to the reducing environment provided by a high-temperature pyrolysis of sucrose into carbon, while the formation of α-Fe_2_O_3_ is promoted by the direct reaction of iron metal with oxygen at 380 °C.^45^ Relevantly, the patterns of the intermediate (α-Fe and pyrolytic C) and the final powder (α-Fe_2_O_3_ and C residue) do not show any reflections of graphite, expected at 2θ values of about 20, 40, and 60°,^7^ thus suggesting the formation of low crystalline carbon in α-Fe_2_O_3_@C. A further insight into the material characteristics is given by the SEM and TEM images of the bare precursor (Figure 2b,d), the synthesis intermediate (Figure 2e,g), and the final sample (Figure 2h,j), respectively. The SEM images reveal that all the materials were formed by the aggregation of submicron primary particles into secondary ones with a size ranging from 1 to 10 μm (Figure 2b,e,h). Despite the similar morphology, the SEM image of α-Fe_2_O_3_@C appears more defined and less bright compared to that of the iron oxide precursor (see Figure 2b,h), likely due to a higher conductivity of the former as compared to the latter. A more defined view of the materials is given by the TEM, which suggests a pristine sample containing almost fully regular γ-Fe_2_O_3_ spherules with an approximate diameter from 50 to 90 nm (Figure 2d). These particles are converted upon Ar-annealing into nano- (80 nm) and sub-micrometrical (500 nm) irregular α-Fe domains enclosed in a thin carbon shell (Figure 2g). This morphology is retained after the final oxidation step to obtain α-Fe_2_O_3_@C (Figure 2j). On the other hand, the EDS analyses actually reveal the complete reduction of the maghemite precursor (Figure 2c) to metallic iron along with the formation of carbon with a weight ratio of 23% during pyrolysis (Figure 2f), which is lowered to about 6% after the final oxidation of the intermediate to α-Fe_2_O_3_@C (Figure 2i), in which all elements are homogeneously distributed (see the corresponding map in the inset of Figure 2i).

According to our previous report on a conversion electrode based on nickel oxide,^35^ we may expect that the morphology observed for α-Fe_2_O_3_@C in Figure 2 would ensure an optimal electric contact between the α-Fe_2_O_3_ grains and buffer volume variation, thus allowing an improved electrochemical process in the Li-cell.^46^ In addition, the encapsulation of nanoparticles into aggregates with a microsize can actually ensure a suitable electrode tap density and, at the same time, mitigate the electrolyte decomposition and increase the cell efficiency.^11,47^

**Figure 3:**
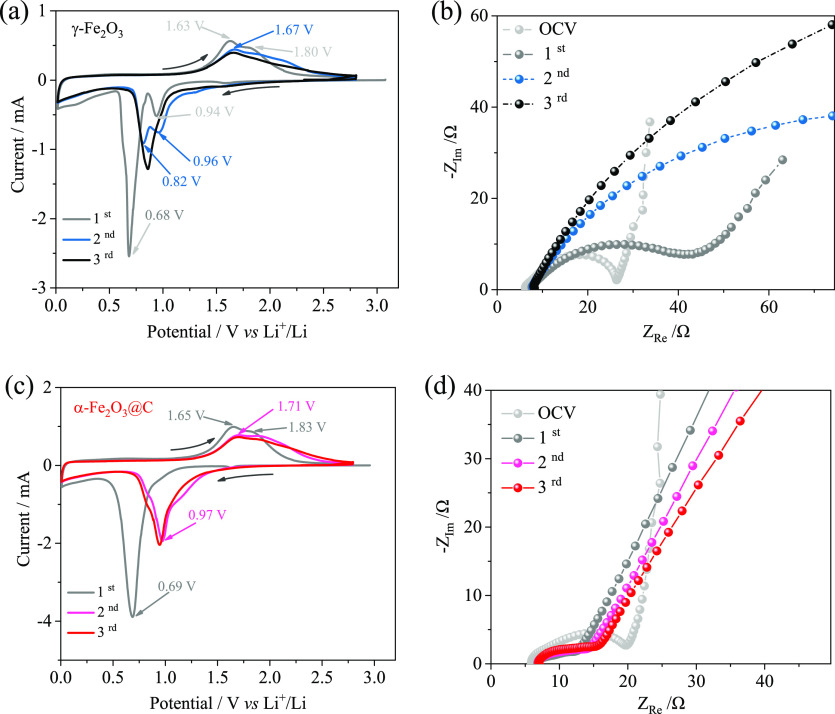
(a,c) CV profiles and (b,d) EIS Nyquist plots in the OCV condition and after 1, 2, and 3 cycles of the (a,b) γ-Fe_2_O_3_ precursor and (c,d) α-Fe_2_O_3_@C composite in T-type cells with lithium disks as counter/reference electrodes and 1 M LiPF_6_ in the EC/DMC (1:1, v/v) electrolyte. CV data collected from 2.8 to 0.01 V vs Li^+^/Li at a scan rate of 0.1 mV s^–1^. Room temperature: 25 °C.

The conversion process of α-Fe_2_O_3_@C is characterized by collecting CV and EIS data (see Figure 3). The CV curve of the γ-Fe_2_O_3_ precursor, which is reported for comparison (Figure 3a), shows during the first discharge a small reduction peak occurring at about 0.9 V versus Li^+^/Li, followed by an intense peak at about 0.7 V versus Li^+^/Li, and a shoulder at low voltage values. Instead, α-Fe_2_O_3_@C (Figure 3c) shows one discharge process at *ca.* 0.7 V versus Li^+^/Li and the low-voltage slope. Hence, the cathodic response of the precursor reflects an initial Li^+^ insertion into the γ-Fe_2_O_3_ structure at low lithiation degrees (peak at 0.94 V *vs* Li^+^/Li in Figure 3a),^48^ the subsequent displacement process at 0.68 versus Li^+^/Li of the oxide to metallic iron enclosed in a Li_2_O matrix,^49^ and the electrolyte decomposition leading to the precipitation of the solid electrolyte interphase (SEI) at low potential values.^11^ Interestingly, α-Fe_2_O_3_@C shows in the first cathodic scan only the displacement process and concomitant SEI formation at 0.69 V versus Li^+^/Li (Figure 3c).

During the first anodic scan, both the γ-Fe_2_O_3_ precursor and the α-Fe_2_O_3_@C electrode have a similar behavior, as they show a broad wave at *ca.* 1.6 V versus Li^+^/Li and a shoulder at *ca.* 1.8 V versus Li^+^/Li, corresponding to the partially reversible conversion process.^49^ The second voltammetry cycle reveals a gradual shift in potential for the reduction processes, namely, to 0.96 and 0.82 V versus Li^+^/Li for γ-Fe_2_O_3_ (Figure 3a) and to 0.97 V versus Li^+^/Li for α-Fe_2_O_3_@C (Figure 3c). On the other hand, during the subsequent cycles, γ-Fe_2_O_3_ and α-Fe_2_O_3_@C undergo different trends, as also suggested by EIS (Figure 3b,d). Accordingly, γ-Fe_2_O_3_ shows a gradual electrochemical deactivation (Figure 3a) as well as a notable increase in the electrode/electrolyte interface resistance, whose value may be extracted from the impedance data at a high-to-middle frequency (Figure 3b). Instead, α-Fe_2_O_3_@C shows a steady-state behavior (Figure 3c), characterized by conversion processes stably leading to intense peaks at about 1.0 V versus Li^+^/Li upon discharging and at about 1.7 V versus Li^+^/Li upon charging, along with an EIS response without any sign of resistance increase (Figure 3d). These observations suggest that the change in the structure and morphology of the iron oxide powder, that is, from γ-Fe_2_O_3_ to α-Fe_2_O_3_@C, greatly benefits the electrode charge transfer.

**Figure 4:**
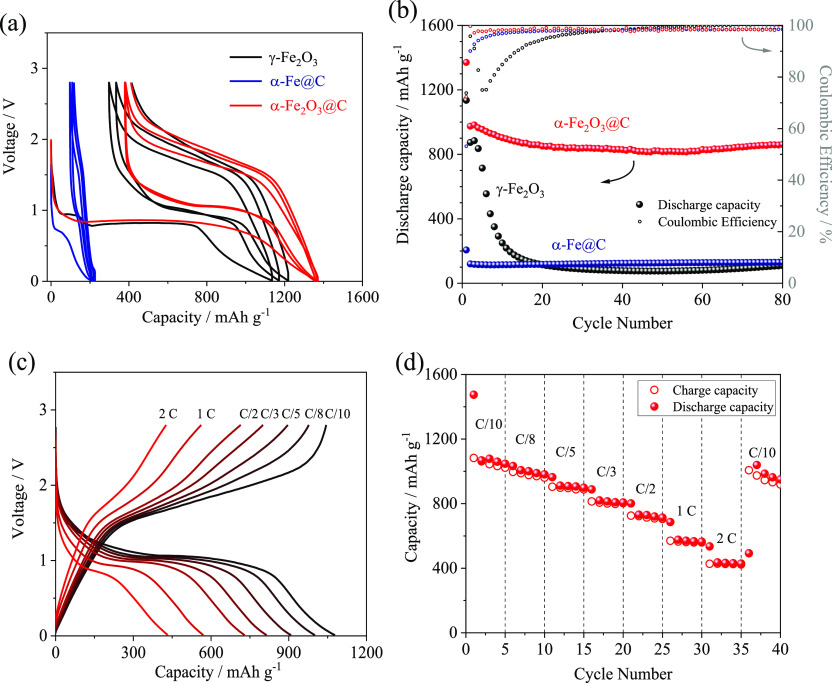
(a) Voltage curves and (b) trends of capacity and Coulombic efficiency at a C/5 rate (1 C = 1007 mA g^–1^) of the γ-Fe_2_O_3_ precursor, α-Fe@C intermediate, and α-Fe_2_O_3_@C composite in coin-type lithium half-cells. (c) Selected voltage curves and (d) trend of specific capacity in a rate capability test of the α-Fe_2_O_3_@C electrode in a coin-type lithium half-cell at C/10, C/8, C/5, C/3, C/2, 1 C, and 2 C rates (1 C = 1007 mA g^–1^). Electrolyte: 1 M LiPF_6_ in EC/DMC (1:1, v/v). Voltage range: 2.8–0.01 V. Room temperature: 25 °C.

The α-Fe_2_O_3_@C is subsequently cycled in a lithium cell at a C-rate of C/5 (1 C = 1007 mA g^–1^) in comparison with the pristine oxide and the synthesis intermediate, as reported in Figure 4a,b. The first discharge evolves mainly around 0.9 V with capacities exceeding the theoretical value for both γ-Fe_2_O_3_ and α-Fe_2_O_3_@C, that is, 1130 and 1370 mA h g^–1^, respectively. The synthesis intermediate, which is formed by inactive iron and disordered carbon, delivers a capacity as low as 190 mA h g^–1^ during the same reduction step, thereby suggesting that the excess capacity observed for the iron oxides could indeed be associated with the SEI formation,^11^ as well as with the possible contribution of the carbon matrix to the lithium exchange in the α-Fe_2_O_3_@C.^46^ During the charge process, the two iron oxides exhibit a sloped voltage signature over 1.7 V, reflecting the typical charge/discharge hysteresis of the Li-conversion electrodes,^15,47^ with the first-cycle capacities of 840 mA h g^–1^ for γ-Fe_2_O_3_ and 970 mA h g^–1^ for α-Fe_2_O_3_@C. Notably, α-Fe_2_O_3_@C delivers a stable capacity upon the 80 cycles considered herein, with an average steady-state value of 860 mA h g^–1^ and almost 100% Coulombic efficiency, while γ-Fe_2_O_3_ shows rapid capacity decay to values as low as 100 mA h g^–1^ after only 20 cycles (see Figure 4b).

The enhanced stability of the α-Fe_2_O_3_@C electrode compared to the pristine γ-Fe_2_O_3_ precursor may be reasonably attributed to an improved electric contact and structural retention upon the discharge/charge cycles provided by the carbon shell already observed in Figure 2, as also suggested by our previous reports.^50^ However, a further beneficial effect of the phase change from γ-Fe_2_O_3_ to α-Fe_2_O_3_ upon the thermal treatments cannot be excluded. In this regard, earlier works have shown that the phase composition may have a significant effect on the behavior of Fe_2_O_3_ in the cell upon long-term cycling.^51^ Moreover, we remark that the morphology certainly affects the electrochemical activity of conversion iron oxides.^52^ The rate capability of the α-Fe_2_O_3_@C electrode is then investigated by performing a cycling test at currents ranging from C/10 to 2 C (Figure 4c,d). Figure 4d indicates a delivered capacity of about 900 mA h g^–1^ at C/10, which decreases to about 800 mA h g^–1^ when the current rate is increased to C/3 due to an expected slight increase in the charge/discharge polarization (Figure 4c). A further increase in C rate to 2 C lowers the reversible capacity to 430 mA h g^–1^, which is a remarkable value considering the high specific current (more than 2 A g^–1^), as compared to the 2 C rate of conventional graphite (0.744 A g^–1^).^7^ Furthermore, as the current rate decreases back to the initial value of C/10 at the 45th cycle, the cell almost recovers the pristine capacity (i.e., about 900 mA h g^–1^), thus accounting for a relevant stability of the α-Fe_2_O_3_@C material by changing the operating conditions. These results suggest that the synthesis pathway adopted herein allows the achievement of an improved α-Fe_2_O_3_@C conversion electrode that might be applied as the anode in a Li-ion battery.^22,35^

**Figure 5:**
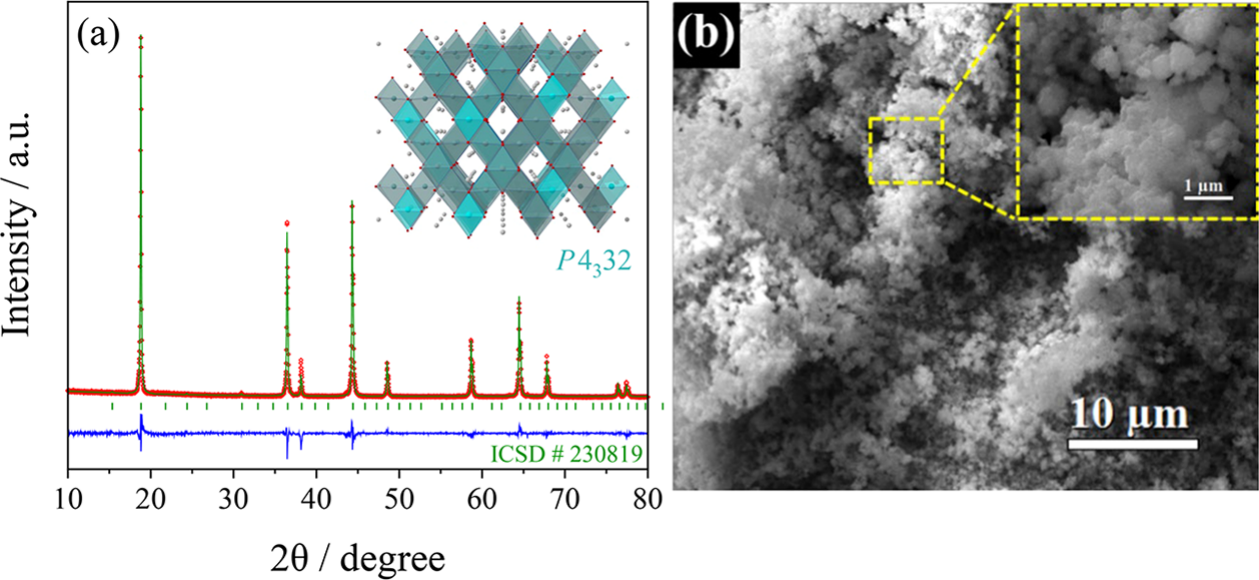
(a) Experimental and refined XRD patterns with reference (ICSD # 230819) and (b) SEM image of LiNi_0.5_Mn_1.5_O_4_ with magnification in the inset.

A LiNi_0.5_Mn_1.5_O_4_ cathode operating in the lithium cell at a voltage as high as 4.8 V is synthesized (Figure 5) and considered the most adequate candidate for achieving a practical application of the Fe_2_O_3_@C conversion anode in a Li-ion battery.^36−38^ The XRD pattern of the cathode powder reported in Figure 5a reveals all the diffraction peaks characteristic of the *P*4_3_32 spinel phase (ICSD # 230819), without signs of impurities, such as those having rock-salt structure (Li_*x*_Ni_1–*x*_O), that cause a deviation from the stoichiometric LiNi_0.5_Mn_1.5_O_4_ composition in *Fd*3̅*m* phases.^53^ Furthermore, the Rietveld refinement of the XRD data indicates a crystallite size of about 100 nm, which is confirmed by the morphological details detected by SEM (Figure 5b). Indeed, the SEM shows nanosized primary particles agglomerated into domains with a microsize, which is considered a suitable configuration for ensuring, at the same time, improved electrochemical activity and limited parasitic electrolyte decomposition.^54^ Nanoparticles can in fact improve the (de)insertion kinetics of lithium into and from the spinel structure of the cathode and concomitantly strengthen the electrolyte oxidation at high voltages due to their relatively high specific surface.^55^ On the other hand, the latter side process may be remarkably suppressed by agglomerating the LiNi_0.5_Mn_1.5_O_4_ nanoparticles into micrometric clusters suitable for achieving a high efficiency in the battery.^56^

**Figure 6:**
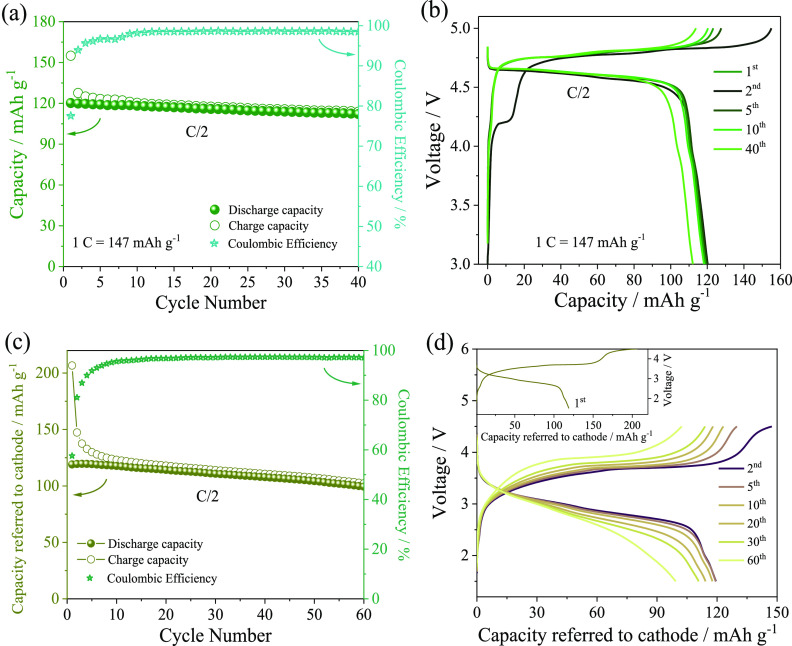
(a) Trends of specific capacity and Coulombic efficiency and (b) selected voltage curves of the LiNi_0.5_Mn_1.5_O_4_ electrode in the coin-type lithium half-cell at a C/2 rate (1 C = 147 mA g^–1^) within 3.0 and 5.0 V. (c,d) Electrochemical response within the 1.5–4.5 V range of the α-Fe_2_O_3_@C/LiNi_0.5_Mn_1.5_O_4_ full cell at a C/2 rate referred to the LiNi_0.5_Mn_1.5_O_4_ cathode mass (1 C = 147 mA g^–1^) in terms of (c) trends of specific capacity and Coulombic efficiency and (d) selected voltage profiles (first cycle in the inset). Electrolyte: 1 M LiPF_6_ in EC/DMC (1:1, v/v). Voltage range: 2.8–0.01 V. Room temperature: 25 °C.

The electrochemical response of LiNi_0.5_Mn_1.5_O_4_ is preliminarily investigated in a lithium half-cell by performing a cycling test at a C/2 rate (1 C = 147 mA g^–1^), as reported in Figure 6a,b. The cathode shows a stable specific capacity of 120 mA h g^–1^ as well as a Coulombic efficiency approaching 100% after few charge/discharge cycles (Figure 6a). We remark herein that LiNi_0.5_Mn_1.5_O_4_ may have either a disordered (space group, *Fd*3̅*m*) or an ordered structure (space group, *P*4_3_32) depending on the presence of oxygen deficiency caused by the above-mentioned rock-salt impurities. In the first arrangement, Ni^2+^ and Mn^4+^ ions are evenly positioned in the 16d and 8a sites, while in the latter one, Ni^2+^, Mn^4+^, and Li^+^ ions are, respectively, distributed in the 4b, 12d, and 8c crystal positions. Notably, Mn^3+^ occurs in the *Fd*3̅*m* framework to balance the oxygen deficiency, thereby leading to a characteristic voltage profile showing a further reversible process at *ca.* 4.0 V versus Li^+^/Li.^53^ In this regard, the half-cell voltage profile (Figure 6b) reveals during the first charge an irreversible plateau at about 4.2 V, most likely attributed to the oxidation of water traces in the electrolyte or undetected Mn^3+^ in the LiNi_0.5_Mn_1.5_O_4_ electrode,^53,57^ as well as a possible Al corrosion.^58^ Furthermore, a capacity excess at high voltages during the first cycle suggests the partial occurrence of the electrolyte decomposition with the deposition of a suitable SEI film, which protects the electrode surface.^59^ Accordingly, the subsequent cycles reveal only the typical high voltage signature of the Ni^4+^/Ni^2+^ redox pair, that is, around 4.7–4.8 V.^60^

It is worth mentioning that LiNi_0.5_Mn_1.5_O_4_-based batteries typically suffer from poor stability at a moderately elevated temperature, showing the degradation of the electrode surface and manganese dissolution in the electrolyte solution. Coating LiNi_0.5_Mn_1.5_O_4_ with thin oxide layers of various properties, such as ZnO, TiO_2_, and Al_2_O_3_, has proven to effectually stabilize the electrode/electrolyte interphase and improve the high-temperature response of the cell.^61−63^ Furthermore, we have recently demonstrated that slight changes in the formulation of the oxalate precursors during the modified co-precipitation synthesis of LiNi_0.5_Mn_1.5_O_4_ may produce a multi-metal spinel framework with enhanced behavior at 55 °C.^53^ These strategies might be suitable for enabling the application of the Fe_2_O_3_@C/LiNi_0.5_Mn_1.5_O_4_ battery above room temperature. The characteristics of this full cell are displayed in Figure 6, which shows the voltage curves (Figure 6c) and the trends of the capacity and Coulombic efficiency (Figure 6d) by referring the specific values to the cathode mass. The α-Fe_2_O_3_@C/LiNi_0.5_Mn_1.5_O_4_ full cell cycled at the constant current rate of C/2 (1 C = 147 mA g^–1^) exhibits a stable response, delivering *ca.* 115 mA h g_cathode_^–1^ during the initial stages of the test and *ca.* 110 mA h g_cathode_^–1^ upon the subsequent cycles, with a Coulombic efficiency approaching 97% (Figure 6c). The related voltage profile during the first cycle (inset of Figure 6d) shows an irreversible profile, possibly ascribed to the above-discussed side processes, that is, structural reorganizations, electrolyte decomposition, and side reactions with the SEI film formation both on the anode and on the cathode.^53,57−59^ Besides, the voltage profiles at the 2nd, 5th, 10th, 20th, 30th, and 60th cycles (Figure 6d) display a steady-state sloped signal centered around 3.2 V, reflecting the conversion process of the α-Fe_2_O_3_@C anode^47^ and the simultaneous (de)insertion of the LiNi_0.5_Mn_1.5_O_4_ cathode^44^ that reversibly occur during the full Li-ion cell operation.^60^ Taking into account the capacity and average voltage values shown in Figure 6c,d, that is, 110 mA h g^–1^ and 3.2 V, respectively, we can estimate for the Fe_2_O_3_@C/LiNi_0.5_Mn_1.5_O_4_ full cell a theoretical gravimetric energy density of about 350 W h kg^–1^, and a practical value approaching 150 W h kg^–1^. Therefore, this battery configuration might outperform similar systems employing a cheap iron oxide conversion anode and cobalt-free spinel cathode.^33^ On the other hand, the conventional graphite/LiFePO_4_ battery may ensure a capacity as high as 150 mA h g^–1^ (when referred to the cathode mass) and operates at *ca.* 3.2 V, thus having a theoretical energy of 480 W h kg^–1^, which might be reflected as a practical value of more than 160 W h kg^–1^.^64^ Notably, this latter system possesses even more interesting features in terms of environmental friendliness and cost due to the absence of cobalt and nickel in the electrode formulation, although the low density of olivine materials adversely affects the volumetric energy of the whole battery.^65^

## Conclusions

An alternative α-Fe_2_O_3_@C conversion anode and a high-voltage LiNi_0.5_Mn_1.5_O_4_ spinel cathode have been synthesized, investigated, and combined in a new Li-ion cell. The anode has been synthesized by a two-step pathway leading to nanoparticles with a uniform and thin carbon shell, while the cathode has been prepared by an alternative approach in the form of nanoparticles agglomerated into microdomains. The α-Fe_2_O_3_@C nanocomposite exhibited excellent electrochemical performance, delivering a capacity that approaches 900 mA h g^–1^, with a stable cycling trend and a suitable rate capability. This simple, fast, and cheap synthesis has therefore been suggested as an advantageous and scalable pathway for obtaining high-capacity conversion metal oxides for battery applications. Moreover, the LiNi_0.5_Mn_1.5_O_4_ cathode revealed a suitable structure and morphology as well as an adequate cycling response in the lithium half-cell, that is, a specific capacity exceeding 120 mA h g^–1^, high efficiency in the first cycle, and an operating voltage of 4.8 V due to the redox activity of Ni^4+^/Ni^2+^. Hence, the combination of the α-Fe_2_O_3_@C and LiNi_0.5_Mn_1.5_O_4_ electrodes led to a full cell which may work at a C/2 rate (referred to the mass of the cathode). This new Li-ion cell delivered a stable capacity of 110 mA h g_cathode_^–1^ at *ca.* 3.2 V with a satisfactory Coulombic efficiency (higher than 97%). Therefore, we estimated a practical energy density approaching 150 W h kg^–1^ and proposed the α-Fe_2_O_3_@C/LiNi_0.5_Mn_1.5_O_4_ battery as a low-cost energy storage system with a moderate environmental impact.
